# CASyM: Chemotype Annotation through Synthesis Mapping

**DOI:** 10.1021/acs.jcim.6c00645

**Published:** 2026-10-15

**Authors:** Paula Torren-Peraire, Jonas Verhoeven, Dorota Herman, Hugo Ceulemans, Igor V. Tetko, Jörg K. Wegner

**Affiliations:** † In-Silico Discovery, Research and Development, 50148 Johnson and Johnson, Beerse 2340, Belgium; ‡ Institute of Structural Biology, Molecular Targets and Therapeutics Center, 364871 Helmholtz Munich—Deutsches Forschungszentrum Für Gesundheit Und Umwelt (GmbH), Neuherberg 86764, Germany; § In-Silico Discovery, Research and Development, Johnson and Johnson, Cambridge, Massachusetts 02142, United States

## Abstract

The identification and annotation of chemotypes is central to structure–activity relationship studies and property optimization in drug discovery. Current computational approaches rely on predefined fragmentation rules or structural similarity to identify these molecular scaffolds, often overlooking the rich information available in reaction data. Here, we introduce CASyM (Chemotype Annotation through Synthesis Mapping), a novel approach that leverages hierarchical reaction data to systematically identify chemotypes within compound libraries. CASyM constructs synthesis graphs from curated reaction data sets, detects major common intermediates, and builds chemotype graphs that capture the hierarchical and interconnected nature of chemical synthesis. SMARTS patterns are extracted to define chemotypes, enabling robust assignment of target molecules, including those absent from the original reaction data. We demonstrate the approach across three case studies using public patent data, proprietary electronic laboratory notebook data from drug discovery projects, and simulated multistep synthesis data for compounds lacking experimental synthesis records. CASyM achieves high coverage of target molecules (85–99%), reveals chemotype-property relationships, and enables chemotype assignment in the absence of experimental data. This reaction-centric approach provides a powerful framework for chemotype annotation, facilitating series exploration and optimization in medicinal chemistry.

## Graphical abstract

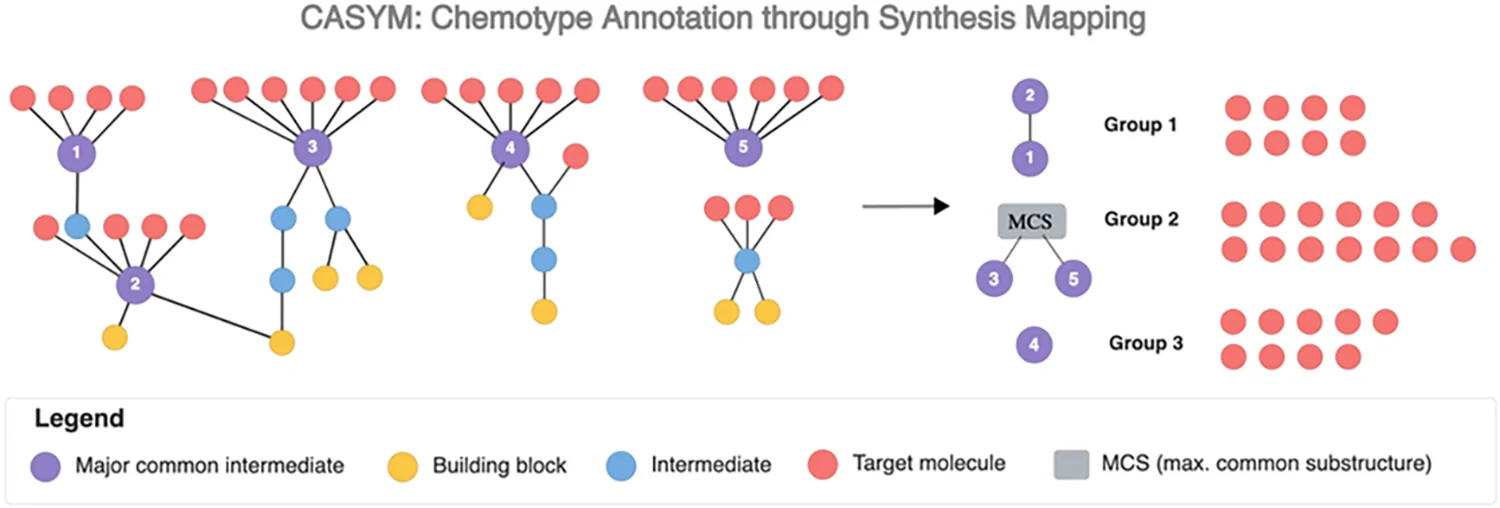

## Introduction

The study of structure–activity relationships and property optimization frequently involves the exploration of peripheral vectors of target compounds, crafted on established structures that are termed chemotypes.^1,2^ Chemotypes can be complex to extract computationally and often rely on expert experience for definition. Understanding the relationship between chemotypes and the respective property space they cover proves vital in drug discovery, as researchers explore and improve the characteristics toward the desired compound space.

Multiple approaches have been developed to address this challenge, focusing on establishing common features within the molecule to subset molecular structures.^3^ This includes the commonly used Murcko scaffolds,^4^ which define a scaffold by removing side chains from a molecule until the assumed core structure is reached, consisting of a combination of ring systems and linkers connecting these. Beyond this, Schuffenhauer et al. developed Scaffold Tree,^5^ a hierarchical system establishing a set of prioritization rules to identify relevant structures across compounds of interest. This was later used as part of Scaffold Hunter,^6^ a suite of tools to visually inspect scaffolds within a compound library based on a variety of chemotype annotation approaches. Lastly, the popular RDKit^7^ package implemented rdScaffoldNetworks,^8^ using a fragmentation process similar to the HierS network^9^ and alternatively the BRICS algorithm^10^ or RECAP.^11^ Generally, all of these approaches focus on a predefined set of logic rules to identify the pertinent scaffold across a set of target molecules. Other approaches instead focus on a clustering approach, based on the structural similarity across compounds, using an unsupervised method to group together relevant scaffolds,^12,13^ or exploring multiple fragmentation and similarity-based clustering algorithms to establish the most beneficial approach to group together relevant chemotypes.^14^ Other methods, such as the OntoChem Chemical Ontology^15^ and ClassyFire,^16^ focus on predefined ontological groups to classify families of compounds based on hierarchical taxonomies; however, these approaches do not capture the synthesis-derived relationships that are demonstrated in this work.

Many of these approaches focus on fragmentation rules, at times defined by retrosynthesis,^17^ yet recover an a priori definition of structure or segmentation. However, there remains an underexploited resource: the reaction data that were used to synthesize the target compounds. These data harbor a wealth of information and have become increasingly prevalent, with multiple methods developed to focus on aspects such as multistep synthesis planning,^18^ single-step retrosynthesis,^19^ reaction conditions,^20^ and yield estimation.^21,22^ These data essentially provide the trajectory of compound evolution throughout the development of a compound series. Although these data have been used to compare and cluster synthesis routes,^23,24^ and retrosynthetic methods have been used to propose potential chemotypes,^17^ no studies have been reported that approach the problem by considering synthesis data to define the chemotypes.

In this work, we utilize the inherently hierarchical nature of reaction data to identify chemotypes across compound libraries, benefiting from common intermediates to extract complex chemotypes. We demonstrate our work and highlight three case studies based on relevant reaction data from internal laboratory notebooks and publicly accessible examples to showcase the advantages of using reaction data to identify chemotypes.

## Methods

### Reaction Data Curation

Starting with project-specific reaction data, we follow a data curation process as described in Torren-Peraire et al.^25^ Projects can be defined as a single-target exploration or across multiple targets. In detail, we use atom-mapped reaction data to identify products and reactants in each reaction, considering all compounds that form part of at least 20% of the final product to be reactants. The data are processed from a retrosynthetic standpoint to create a directed graph where leaf nodes are compounds that did not need to be previously synthesized. Root nodes are compounds where a synthesis ends and are the starting points of the synthesis graphs. From the synthesis data, these nodes are compounds that were not explored further and, therefore, are candidate target molecules. However, due to the complexity of synthesis data, it cannot be assumed that all root compounds are target molecules. Importantly, we deviate from Torren-Peraire et al.^25^ to assess a project as a whole, rather than focusing on the individual convergent routes, not discarding any synthesis graphs that contain cycles or are only associated with a single-target molecule. Each weakly connected component of the graph is termed a synthesis graph, where a single project can contain multiple synthesis graphs.

### Chemotype Annotation

With the project-specific synthesis graphs, we identify those that contain common intermediates and compounds that are used in the synthesis of multiple root compounds. For each synthesis graph, we identify the number of root compounds to which each intermediate is associated. A threshold of root compounds per common intermediate is set, and synthesis graphs that do not contain at least one common intermediate above the threshold are discarded (Figure 1). The threshold is set by the user; specific details on the setting used for the highlighted case studies can be found in the Supporting Information. Different synthesis graphs contain differing numbers of root compounds; therefore, the threshold is set dynamically to reflect the size of the synthesis graph. The threshold for setting an intermediate as a common intermediate (*c*) is defined as

$$c=\min\left(\left[\frac{t}{m}\right],n\right)$$

where *t* is the total number of root compounds in the synthesis tree, *m* is the minimum number of root compounds per intermediate, and *n* is the upper limit for the number of root compounds. Both *m* and *n* are hyperparameters that can be set at initialization. By default, any synthesis graph that contains only one root compound is discarded.

**Figure 1:**
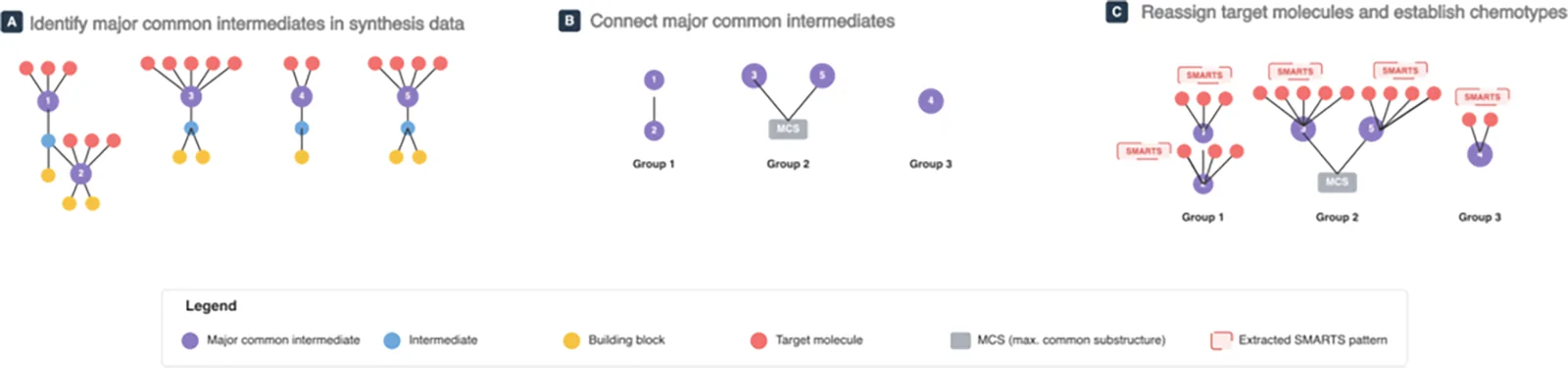
CASyM method figure depicting the process from identifying major common intermediates to developing the chemotype graph. (A) Major common intermediates are identified within the synthesis data. (B) Major common intermediates are isolated into a chemotype graph, where they are relinked if they share a reaction path or share a maximum common substructure. (C) Target molecules are relinked to the major common intermediates, and SMARTS patterns are extracted based on the molecules for each major common intermediate. Each group is now referred to as a chemotype.

Considering the filtered synthesis graphs, major common intermediates are identified and serve as the basis for the identification of chemotypes. For each synthesis graph, nodes that have a path to at least *c* root nodes, i.e., are involved in the synthesis of at least *c* root compounds, are flagged as potential major common intermediates. If a sample of target molecules is provided, the potential major common intermediates are filtered such that all major common intermediates are compounds that have a reaction record to at least *c* number of target molecules. These major common intermediates are introduced as nodes into a new graph instance, termed the chemotype graph.

In some cases, there may be extensive exploration of a series within a synthesis graph (Figure 1). As such, within the chemotype graph, a directed edge is introduced between common intermediates, which are connected by *r* or fewer reaction steps. The directed edge is established in the same retrosynthetic direction as in the synthesis graph. For example, if in the synthesis graph, compounds A and D are identified as major common intermediates, and the synthesis graph has the following reactions: A → B.C; C → D, then a directed edge is created between nodes D and A within the chemotype graph, as exemplified in nodes 1 and 2 in Figure 1. A similarity threshold, *s*, can be set where major common intermediates are linked based on their maximum common scaffold, which covers at least half of both compounds. These may only be linked in the case that both major common intermediates are at most two reaction steps from the root compounds. In this case, a dummy node is added to the chemotype graph, with a directed edge from the dummy node to each compound, as shown on nodes 3 and 5 in Figure 1. Separate chemotypes are defined based on the weakly connected components of the chemotype graph. Each collection of connected nodes and edges is considered a chemotype, with each individual component considered a separate chemotype. Moreover, the hierarchical nature of this can be used to further define levels within the chemotype.

Once the chemotypes are established within the chemotype graph, the root compounds from the synthesis graphs are reconnected to the chemotype graph. Root compounds are assigned to the most advanced major common intermediate based on route depth, with which they share a path in the synthesis graph. This means that an edge is added between the closest major common intermediate, as defined by the number of reaction steps, and the root compound. In the case that a root compound has an equal number of steps to different common intermediates and different chemotypes, then the root compound will have an edge to both common intermediates and, therefore, both chemotypes. The process is carried out for all root compounds. In the case that none of the major common intermediates within a chemotype are associated with a root compound, the chemotype is discarded. After carrying out the process for all root compounds, a SMARTS^26^ pattern is extracted for each major common intermediate based on the maximum common substructure shared across the compounds. The SMARTS pattern is based on the root compounds directly associated with the major common intermediate, and the SMARTS pattern must cover at least 20% of all of the associated root compounds to be considered, ensuring that the SMARTS pattern covers beyond a singular reactant. Additionally, the pattern must contain a minimum of 10 atoms, avoiding small building blocks from being used to define the common substructure. Additionally, if a set of compounds of interest is provided at initialization, then an additional SMARTS pattern is created, encompassing only the root compounds flagged as compounds of interest. This provides a collection of SMARTS patterns per chemotype, with at least one SMARTS pattern associated with each major common intermediate that is associated with root compounds. We summarize all key terms in Table 1.

**Table 1:** Definition of Key Terms Used in This Work

term	definition
Common intermediate	A compound that lies on the synthesis path of more than one root compound within a single synthesis graph.
Major common intermediate	A common intermediate that lies on the synthesis path of at least c root compounds and is used as the anchoring compound for a subgroup of molecules. One or more major common intermediates, together with the edges between them, constitute a chemotype.
Chemotype	A subgroup of molecules sharing a common chemical definition. In this work, a chemotype is derived from synthesis history, not from the structure of the target molecules alone.
Scaffold	A definable chemical substructure that allows a group of compounds to be a subset. Used here in its established, rule-based sense (e.g., a Murcko scaffold), i.e., derived from the structure of a molecule alone.

### Target Molecule Assignment

Once the chemotype graph has been developed, this can be used to assign chemotypes to compounds of interest, referred to as target molecules. Considering the chemotype graph, target molecules can be assigned to each major common intermediate and, therefore, the chemotype. This is carried out by comparing the target molecule to each major common intermediate SMARTS pattern. At this point, there are two potential options: either selecting the SMARTS pattern that has the greatest coverage over the target molecule, essentially selecting the most advanced common intermediate. Alternatively, the SMARTS pattern with the lowest score, which is calculated as the proportion of the target molecule covered by the SMARTS pattern, ensuring a minimum coverage greater than a reactant, is then divided by the number of major common intermediates in the chemotype. In this way, we minimize the number of chemotypes while also avoiding overly specific SMARTS patterns. Moreover, compounds associated with a major common intermediate can be flagged as having a reaction precedent for their links. All assigned target molecules can be added to the chemotype graph using an edge between the identified major common intermediate and the target molecule. In both cases, exactly one SMARTS pattern, and therefore exactly one chemotype, is retained per target molecule, so the assignments reported in this work are unique by construction, and no target molecule is counted under more than one chemotype. Where a target molecule matches SMARTS patterns belonging to several chemotypes, the selection criterion described above resolves the ambiguity. Target molecules matching no SMARTS pattern are left unassigned, as reflected in the coverage figures reported in Table 2.

**Table 2:** Summary of Coverage, Number of Clusters, and Number of Majority Clusters across All Three Case Studies^a^

case study	number of target molecules	coverage (%)	number of chemotypes	number of major chemotypes
1	2861	85.0	23	5
2	1271	94.7	17	5
3	298	99.3	4	2

a Coverage refers to the percentage of target molecules that are assigned to chemotypes. The total number of chemotypes and the number of major chemotypes, which are associated with at least 5% of target molecules, are reported.

### Data

Through the three case studies, all target molecules used are patented compounds and are available in the public literature. All case studies use target molecules from multiple patents to increase the potential complexity and the number of featured chemotypes. Case study one is based on reactions extracted from US patents, as described by Lowe.^27,28^ In case study two, the reaction data used is based on Johnson and Johnson Electronic Laboratory (J&J ELN) data as described in Neves et al.^29^ In both data sources, reactions are extracted using either US patent numbers or internal project identifiers as identifiers to ensure that the reaction data used are specific to only the target of interest. For case study one, all documents from USPTO were parsed to extract synthesis graphs,^25^ and documents with at least 500 root compounds were selected. Potential documents were filtered such that the final selection contained at least one product in common with at least one other document, ensuring a more complex test case instead of uniquely defined synthesis graphs. Filtering led to a total of 9 USPTO grant documents selected. When running the case study, the full patent information was used, not only the synthesis graph that was used to select the documents.

Lastly, case study three uses the multistep synthesis approach from Torren-Peraire et al.^25^ using the model trained on USPTO data to carry out the search. The raw search is used before convergent route extraction, instead of focusing on any proposed reactants that have 10 or more heavy atoms. Details on the settings used can be found in Supporting Table 2.

## Results and Discussion

To showcase the approach, we delve into three case studies that highlight the benefits of a reaction-focused chemotype-annotation approach.

### Case Study One: Use of Public Data

The USPTO^27,28^ data set is a collection of reactions that contains a corpus of over 3 M reactions spread across patented projects. These data are widely available and contain reactions from 40 years of patent applications and grants. We tested CASyM on USPTO patent data to assess the ability to distinguish between chemotypes across projects in readily available synthesis data. Using 9 documents from the USPTO data set,^30–37^ we parse all documents conjointly as a singular project; details on the selection of these documents are described in Methods. All compounds that are not synthesized further within the synthesis graphs are assumed to be target molecules, leading to 2861 target molecules. With this, 23 chemotypes are identified across the patents (Table 2), highlighting recurring scaffolds within the projects, with 5 chemotypes accounting for 62.6% of all patented target molecules (Figure 2 and Supporting Figure 1). Given the chemotype graph, we can further explore the interconnections of synthesis data, highlighting 72 major common intermediates identified across the projects used in the five most populated groups.

**Figure 2:**
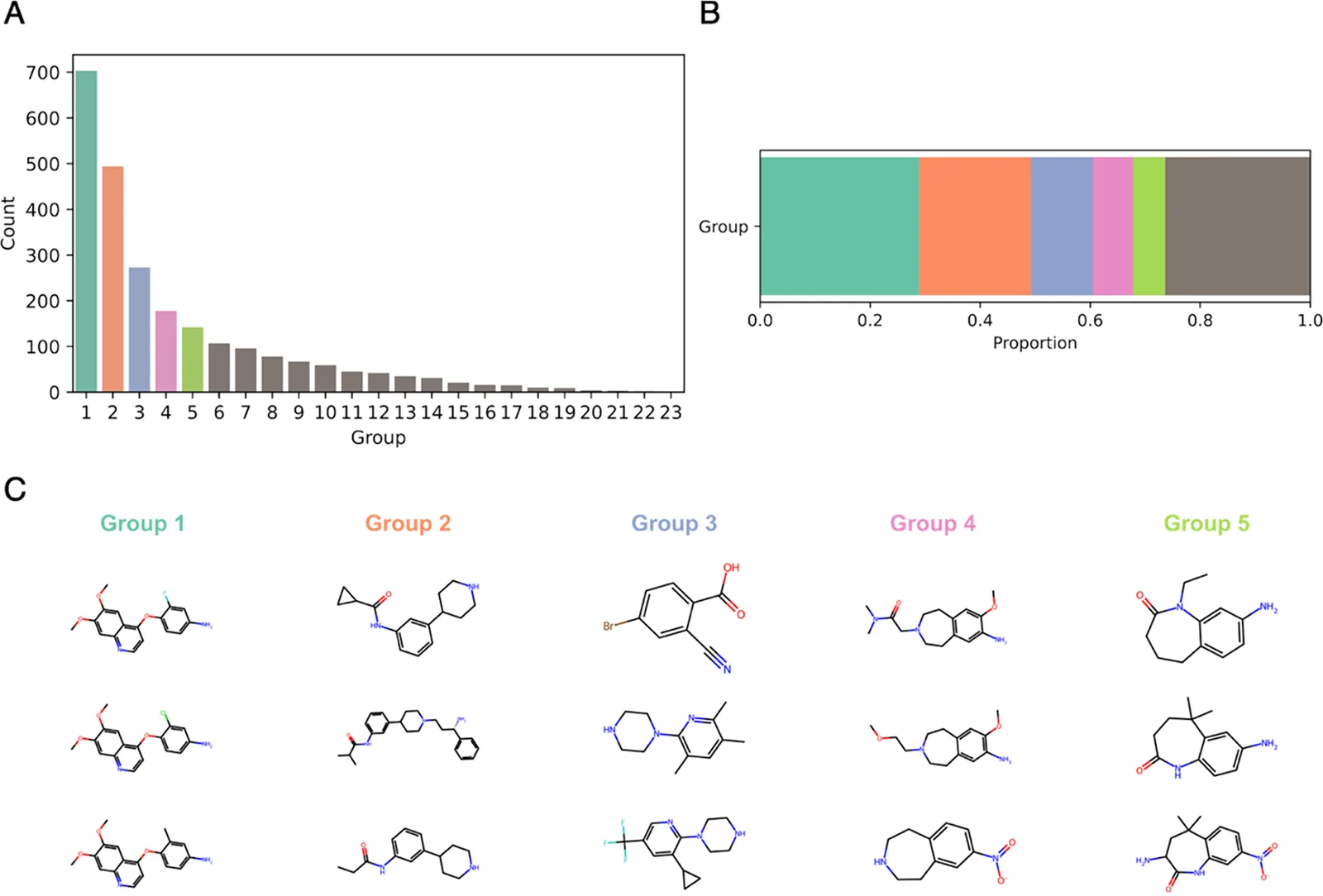
Case Study One: Distribution of identified chemotypes (A) and number of target molecules associated with each (B). Examples of major common intermediates from the five most populated chemotype groups, illustrating the structural diversity within each CASyM-defined group (C). The full structure of major common intermediates is shown in Supporting Figure 1.

Using these data, we can distinguish overlapping chemotypes that may be present across multiple patents and explore advanced common intermediates that are linked to these. Across the five major chemotypes, there are typically two layers of common intermediates, initially a more general structure, which then expands into numerous, more complex, major common intermediates that are, in turn, associated with target molecules. We see a high prevalence of MCS nodes, given the heterogeneity found in both multiple patent documents and the synthesis data itself. This allows us to further group seemingly unconnected synthesis graphs that may deviate by only minimal differences. In fact, groups 1, 3–5 are combinations across at least two patent documents, whereas group 2 is made up of a singular patent with a singular main synthesis graph.

To place CASyM in context with established grouping methods, we compared the chemotype assignments from Case Study 1 against three structure-based approaches applied to the 2454 molecules assigned to a chemotype by CASyM. Murcko scaffold decomposition yielded 642 unique scaffolds, of which 359 (56%) were singletons grouping only a single molecule, with only 283 scaffolds containing two or more members, representing 28-fold more groups than CASyM’s 23 chemotypes. The rdScaffoldNetwork algorithm, which generates a full hierarchy of ring-reduced scaffolds, produced 4481 nodes with 2450 root-level scaffolds for the same 2454 molecules, resulting in no single interpretable classification level analogous to chemotypes. Fingerprint-based Butina clustering using ECFP4 fingerprints at a Tanimoto similarity threshold of 0.4 yielded only 51 clusters, with the largest single cluster containing 697 molecules (28% of the entire set), reflecting the inability of fingerprint similarity at this threshold to resolve synthetically distinct series. Increasing the threshold to 0.6 produced 296 clusters with 108 singletons—13-fold more groups than CASyM—and highly unbalanced cluster sizes. In contrast, CASyM’s 23 chemotypes are all populated (no singletons), balanced, and defined by shared synthetic paths rather than 2D structural overlap, making them directly interpretable in the context of the reaction data from which they derive.

### Case Study Two: Use in Drug Discovery Projects

Typically, researchers work on multiple series simultaneously, exploring each series as it progresses. Therefore, having the ability to clearly distinguish among chemotypes is important. Computer-aided chemotype identification from synthesis data can allow medicinal chemists to further explore and optimize the molecular properties of specific series without having to manually annotate the chemotypes present in projects. With CASyM, we can identify varying chemotypes across target molecules and even assign molecules absent from the reaction data to chemotypes. Using 1271 patented target molecules from a target of interest, we identify a chemotype for 94.7% of target molecules, across 17 chemotypes (Table 2 and Figure 3A).

**Figure 3:**
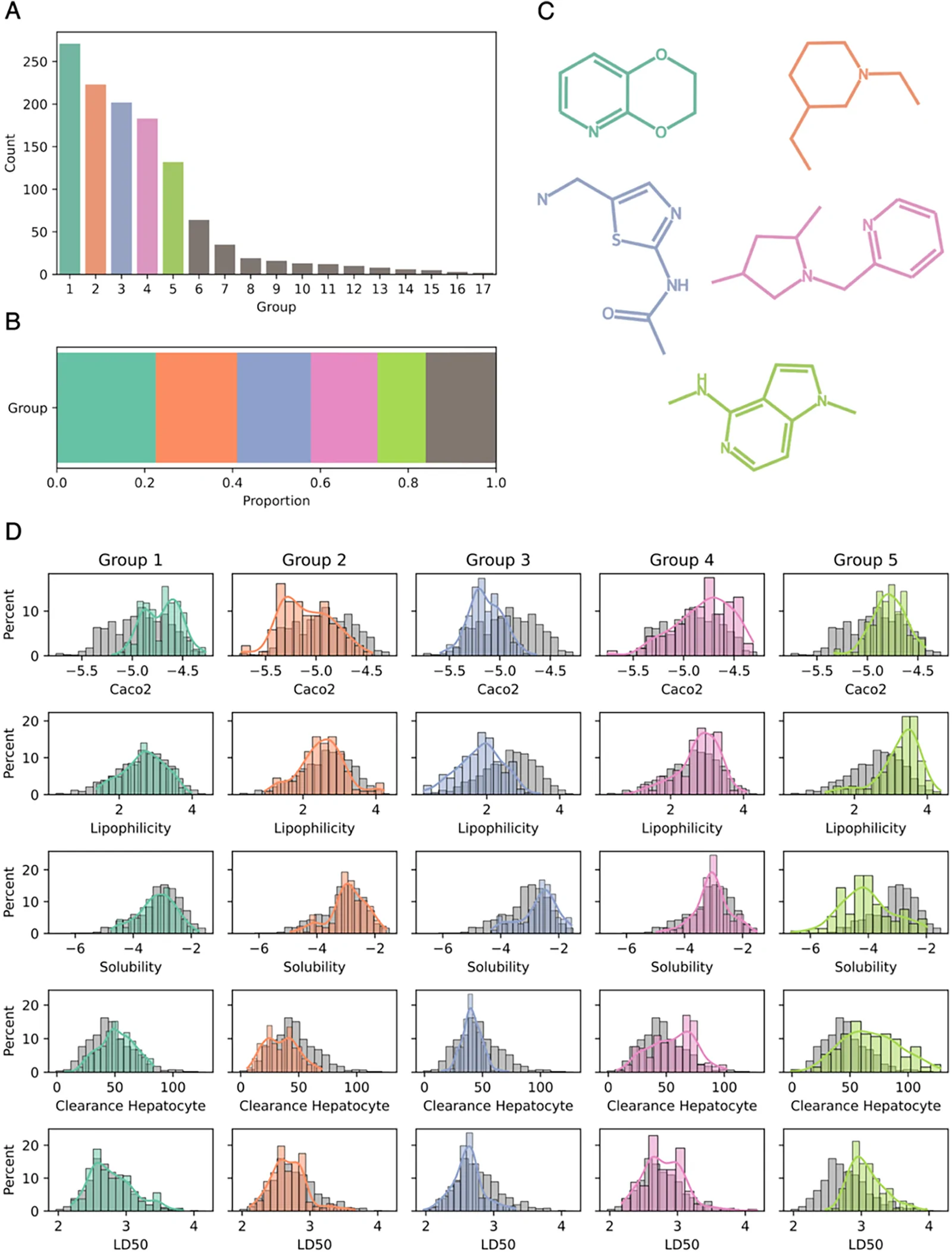
Case Study Two: Distribution of identified chemotypes (A) and proportion of target molecules associated with each (B), highlighting main substructures which identify each of the chemotypes (C). Distribution of suggested molecular properties as computed by ADMET-AI, gray distribution shows background distribution, distributions in color refer to compounds which specifically form part of that grouping (D). Colors are shared across the figure.

Focusing on the top five chemotypes, which make up 79.5% of all target molecules (Figure 3B), we can further explore the potential ADMET properties of these. Using publicly available molecular property models,^38^ we showcase the differing properties of the target molecules within each chemotype (Figure 3C,D). There are apparent differences between the background distribution that could be assumed when assessing all compounds jointly and the suggested properties of each chemotype group. For example, despite group 1 being the most populated group, we see a skew in the distribution of Papp with Caco-2, showing a higher permeability than the other chemotype groups. In contrast, group 3 has a noticeably lower permeability and lipophilicity. This trend continues across the chemotypes, where each group shows different distributions across the highlighted properties.

The property distributions in Figure 3 reveal synthesis-aware distinctions between groups, where each of the five primary chemotype groups is statistically distinct from the background distribution of all remaining compounds (Kolmogorov–Smirnov test, all *p* < 0.005). Group 1, the most populated group, shows higher predicted permeability than the background (KS = 0.53, *p* = 3.2 × 10^–67^; median Caco-2 log *P*_app_ = −4.79 vs −4.94), while Group 2 shows markedly lower permeability (KS = 0.51) and the most distinct lipophilicity profile (KS = 0.44, median log *D* = 1.72 vs 2.32 background). Group 4 stands out as a distinctly more lipophilic series (KS = 0.53, median log *D* = 3.17). Compared to Murcko scaffold grouping (top-5 scaffolds covering 12% of compounds) and ECFP4 fingerprint clustering (top-3 clusters covering 36%), CASyM provides substantially broader coverage (82%, all 5 groups statistically significant) with consistent property discrimination across chemotypes, confirming that synthesis-aware grouping captures chemically meaningful series.

### Case Study Three: Beyond Available Reaction Data

There may be cases in which reaction data for compounds of interest are not readily available. In this case, it is possible to carry out a simulation of multistep synthesis data to then use chemotype annotation in place of experimental multistep synthesis data. As such, considering 298 patented target molecules from a target of interest, we use a computational convergent route multistep synthesis planning approach^25^ to propose links across the target molecules. Using the simulated reaction data, we run the CASyM pipeline to annotate relevant chemotypes. CASyM assigns a chemotype to 95.6% of target molecules across six chemotypes (Figure 4), which can be compared to the experimental reaction data, where we assign a chemotype to 99.3% of target molecules, with two groups making up 97.8% of all assigned molecules. Interestingly, there is a greater degree of fractioning across the groups from the convergent search data as compared to the experimental synthesis data. Group 1 from the synthesis data is mostly found in groups 2–4, whereas group 2 is found in groups 1 and 5 from the convergent search. Despite some additional fractioning, the approach can establish chemotypes, despite no prior synthesis data being available. This enables the use of the convergent search approach together with the CASyM pipeline as a proxy in the case that novel compounds are explored but do not yet have recorded synthesis data, such as through de novo compound generation. The exploration demonstrated in this case study is inherently dependent on the multistep synthesis planning method employed, as different retrosynthetic algorithms may produce different proposed routes. However, the consensus over multiple target molecules provides robustness: even if individual routes differ between planning methods, the underlying target molecules remain the same and the resulting chemotype groups should remain stable across reasonable variation in route generation. The specific major common intermediate identified may vary between methods, but this represents an open area for further investigation into how the choice of the synthesis planning algorithm influences chemotype definition at scale.

**Figure 4:**
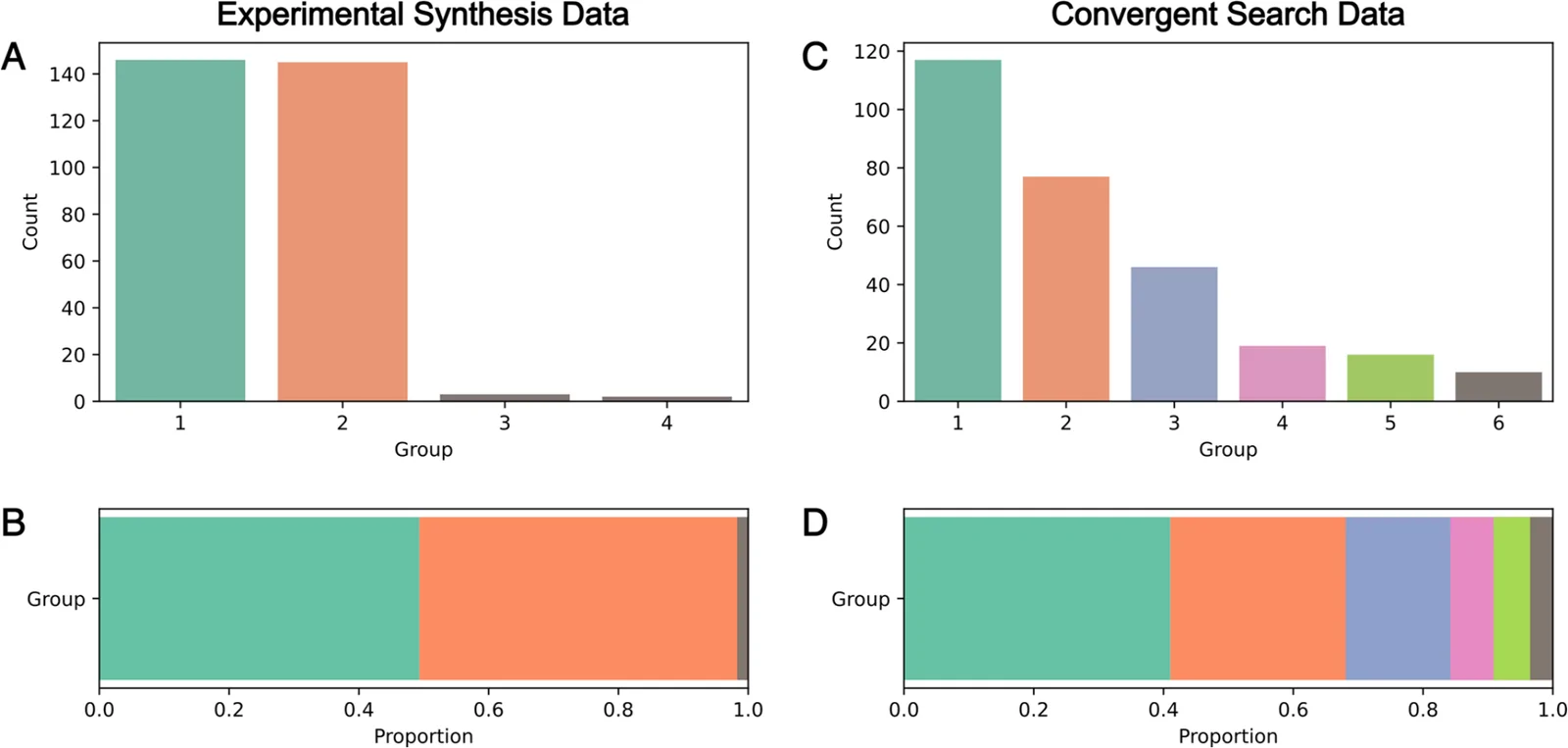
Case Study Three: Distribution of identified chemotypes (A, C) and proportion of target molecules associated with each chemotype (B, D) for experimental synthesis data approach and approximating synthesis data using the convergent search approach.

### Comparison to Established Methods

CASyM proposes an inherently distinct method for establishing compound chemotypes. The method does not rely on pre-established fragmentation, ontology, or order of addressing compound atoms or bonds; instead, CASyM focuses on the identification of chemotypes based on reaction data, be it from historical or simulated sources. This allows the method to propose chemotypes that are relevant to project teams or drug discovery teams based on the explorations that have been carried out therein. Oftentimes, the definition of chemotypes is not solely based on a specific structure but instead on interaction types that may be present and facets that cannot be captured by current methods. This is further exemplified by the results highlighted in case study 1 compared to commonly used approaches within cheminformatics.

Importantly, this method is not intended for massive library annotation of compounds; for this, we defer to other well-established methods. Because it relies on recorded or simulated synthesis data, CASyM produces information-rich chemotypes at the scale of projects, but it is not intended for the annotation of large virtual libraries for which structure-derived methods remain the appropriate choice.

## Conclusion

Chemotype and scaffold annotation can largely enable hit exploration by allowing a better understanding of the characteristics of molecules within a project of interest. However, chemically relevant chemotype annotation can prove challenging for computational methods because of the sheer complexity and nuance of chemotypes across projects of interest. In this work, we propose CASyM, a tool that allows for the exploration and annotation of relevant chemotypes by utilizing synthesis data to produce relevant chemotypes. The novel approach identifies chemotype graphs, made up of major common intermediates, which benefit from the inherent hierarchy to further explore the synthesis data. The approach allows the identification of chemically relevant chemotypes, avoiding the fractionation of related structures over multiple groups. We describe the work through three case studies, based on public synthesis data, electronic laboratory notebook data, and examples beyond available data.

## Supplementary Material



## Data Availability

The CASyM code is available at https://github.com/aidd-msca/CASyM/tree/main. The public USPTO reaction data used in Case Study 1 and the analysis scripts are provided together with an explainer on how to use them.

## Notes

Additional information regarding the settings used for CASyM and the convergent routes search (PDF)
