# Supplementary material for: CASyM: Chemotype Annotation through Synthesis Mapping
Source: J Chem Inf Model. 2026 Oct 15;66(20):13609–17. doi: 10.1021/acs.jcim.6c00645 (PMC13715518; doi:10.1021/acs.jcim.6c00645)
Supplement: Supplementary file 1 [file ci6c00645_si_001.pdf]

Supporting Information

CASyM: Chemotype Annotation Through Synthesis Mapping

Paula Torren-Peraire[1,2], Jonas Verhoeven[1], Dorota Herman[1], Hugo Ceulemans[1], Igor V. Tetko[2] & Jörg K. Wegner[3]

1 In-Silico Discovery, Research & Development, Johnson & Johnson, Beerse, 2340, Belgium

2 Institute of Structural Biology, Molecular Targets and Therapeutics Center, Helmholtz Munich – Deutsches Forschungszentrum für Gesundheit und Umwelt (GmbH), Neuherberg, 86764, Germany

3 In-Silico Discovery, Research & Development, Johnson & Johnson, Cambridge, 02142, US

**Corresponding Author: Jonas Verhoeven; Email: jverhoe9@its.jnj.com*

Contents:

Supplementary Table 1: Default CASyM settings used for all case studies.

Supplementary Table 2: Convergent route search settings.

Supplementary Figure 1: Full structure of major common intermediates used to define the five most populated chemotypes from Case Study One.

Supplementary Figures & Tables

Supplementary Table 1: Default CASyM settings used for all case studies.

**Parameter**	**Value**
Chemotype Steps	4
Common Intermediate Minimum connections (m)	3
Common Intermediate Maximum connections (n)	5
Similarity Threshold	0.8
Lowest Score	True

Supplementary Table 2: Convergent route search settings.

**Parameter**	**Value**
Number of nodes explored per iteration (k)	128
Max time	60s
Max iterations	300
Max route length	4
Building block set	eMolecules (33M building blocks)

Group 1

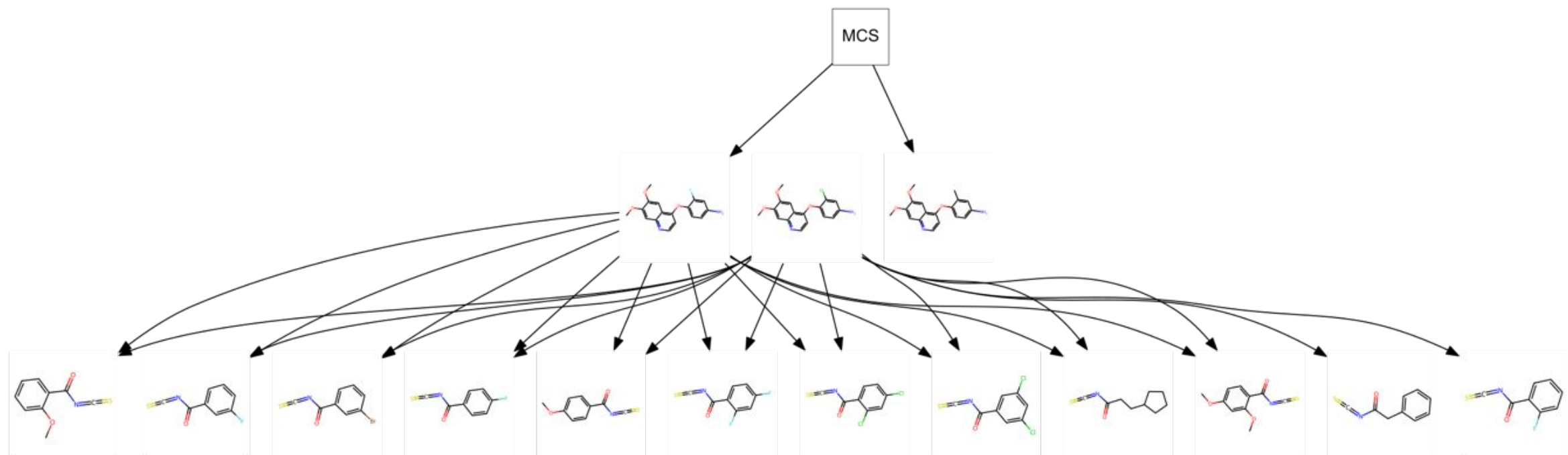

Group 2

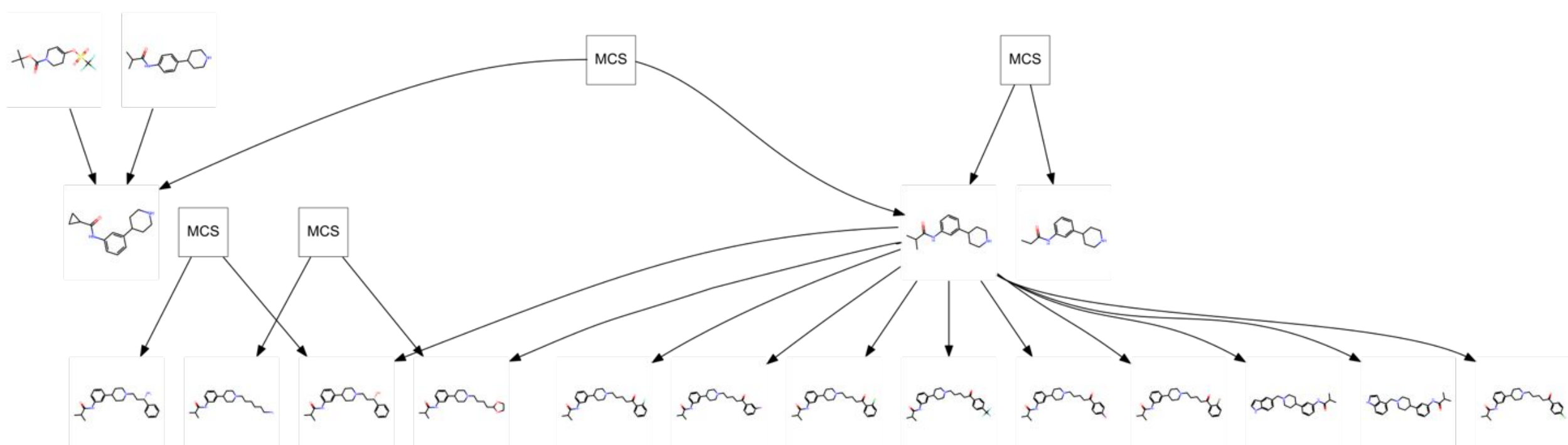

Group 3

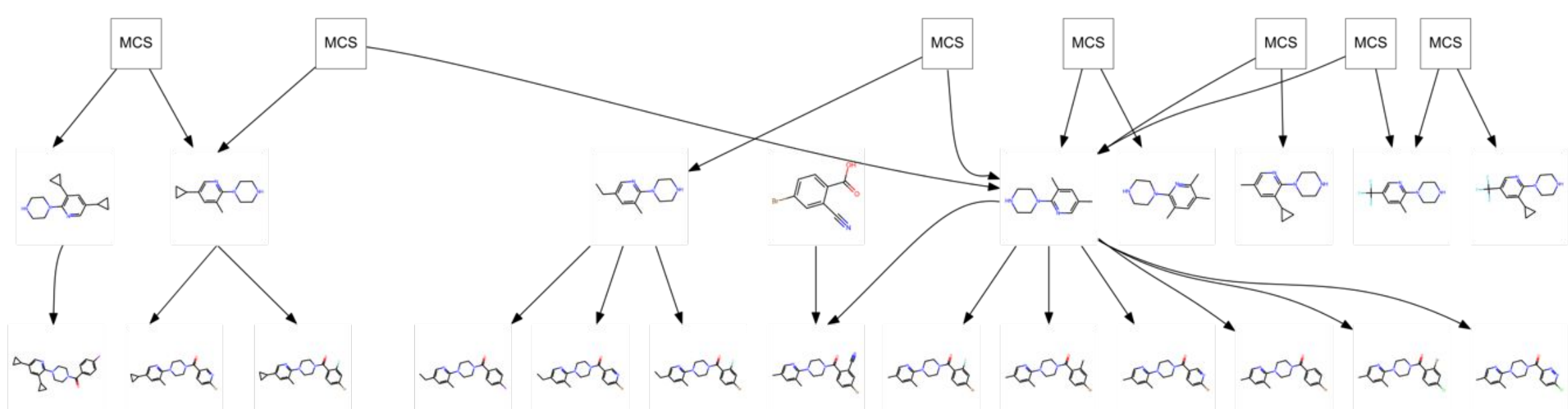

Group 4

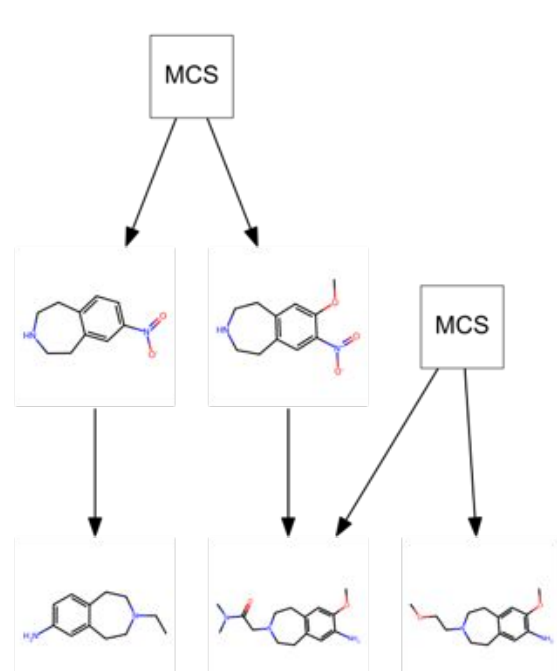

Group 5

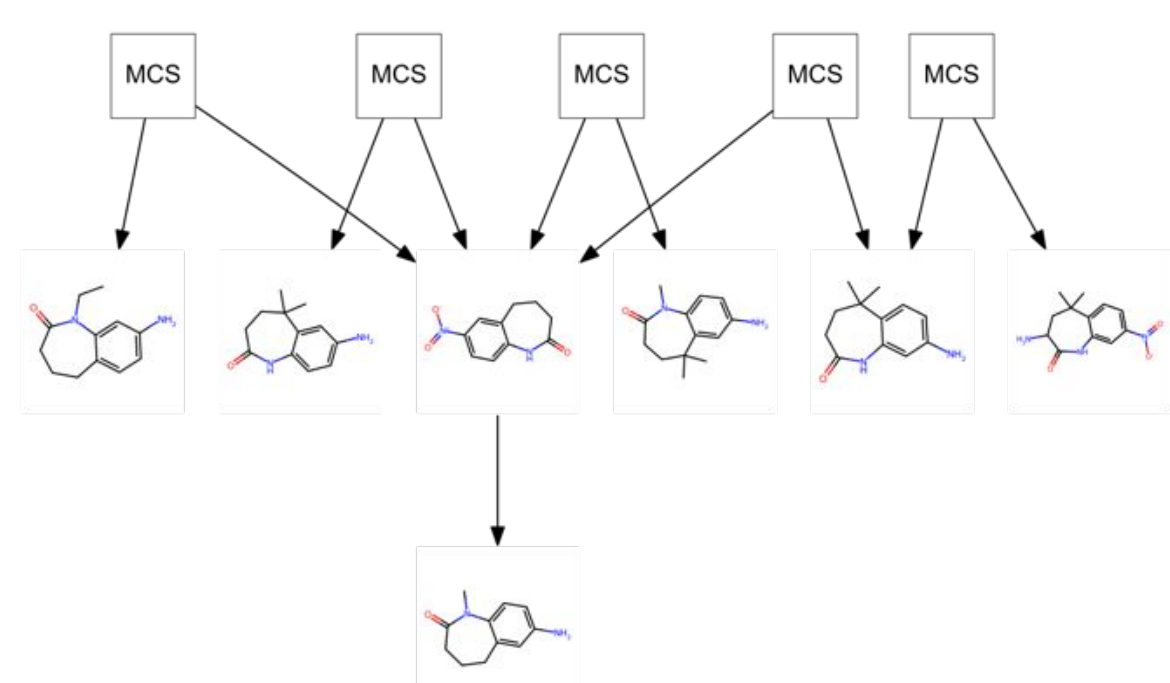

Supplementary Figure 1: Full structure of major common intermediates used to define the five most populated chemotypes from Case Study One. MCS refers to major common intermediates that are linked through maximum common substructure. For visualization purposes, some common intermediates were removed from Groups 2 and 3, pertaining only to highly populated predecessors.
